# Flexible parsing, interpretation, and editing of technical sequences with *splitcode*

**DOI:** 10.1093/bioinformatics/btae331

**Published:** 2024-06-14

**Authors:** Delaney K Sullivan, Lior Pachter

**Affiliations:** UCLA-Caltech Medical Scientist Training Program, David Geffen School of Medicine, University of California, Los Angeles, Los Angeles, CA 90095, United States; Division of Biology and Biological Engineering, California Institute of Technology, Pasadena, CA 91125, United States; Division of Biology and Biological Engineering, California Institute of Technology, Pasadena, CA 91125, United States; Department of Computing and Mathematical Sciences, California Institute of Technology, Pasadena, CA 91125, United States

## Abstract

**Motivation:**

Next-generation sequencing libraries are constructed with numerous synthetic constructs such as sequencing adapters, barcodes, and unique molecular identifiers. Such sequences can be essential for interpreting results of sequencing assays, and when they contain information pertinent to an experiment, they must be processed and analyzed.

**Results:**

We present a tool called *splitcode*, that enables flexible and efficient parsing, interpreting, and editing of sequencing reads. This versatile tool facilitates simple, reproducible preprocessing of reads from libraries constructed for a large array of single-cell and bulk sequencing assays.

**Availability and implementation:**

The *splitcode* program is available at http://github.com/pachterlab/splitcode.

## 1 Introduction

The reads that result from next-generation sequencing libraries can contain many types of synthetic constructs, or technical sequences, including adapters, primers, indices, barcodes, and unique molecular identifiers (UMIs) (Kivioja *et al.* 2011, Martin 2011, Kebschull and Zador 2018, Melsted *et al.* 2019, Johnson *et al.* 2023, Booeshaghi *et al.* 2024). These oligonucleotide sequences are defined by the technicalities of sequencing-based assays and experiments, with each sequence being either a completely unknown sequence, a known sequence, or an unknown sequence that is a member of a set of known sequences. There are many read preprocessing tools for editing and extracting information from such sequences, including the widely used tools cutadapt (Martin 2011), fastp (Chen *et al.* 2018), and Trimmomatic (Bolger *et al.* 2014) for adapter and quality trimming, UMI-tools (Smith *et al.* 2017), and zUMIs (Parekh *et al.* 2018) for UMI processing, BBTools (https://sourceforge.net/projects/bbtools/) (Bushnell *et al.* 2017), and reaper for more general filtering operations, INTERSTELLAR for read structure interpretation (Kijima *et al.* 2023), Picard (https://github.com/broadinstitute/picard), and fgbio (https://github.com/fulcrumgenomics/fgbio) for many read manipulation operations, among many other tools (Kong 2011, Roehr *et al.* 2017, Liu 2019, Battenberg *et al.* 2022, Cheng *et al.* 2024). Many of these tools define a “read structure” to describe the layout of a read; e.g. fgbio uses a sequence of <number><operator> operators where the number of the length of a segment and the operator describes how the segment should be processed. However, no one tool can adequately address all technical sequence preprocessing tasks. Some methods, such as adapter trimming methods, can only remove identified technical sequences from reads but lack the ability to store information about technical sequences that are relevant to the provenance of the read. Other methods can extract and store technical sequences from reads but are limited to only extracting sequences at defined positions of defined lengths within reads, and may present limited options for handling variable position and variable length segments. Still other methods are designed for only a specific type of assay, such as single-cell RNA-seq. Technologies such as (long-read) SPLiT-seq (Rosenberg *et al.* 2018, Rebboah *et al.* 2021), SPRITE (Quinodoz *et al.* 2018, 2022), and Smart-seq3 (Hagemann-Jensen *et al.* 2020), contain complex, multifaceted technical sequences that currently are processed by custom scripts or specific use-case modifications to existing tools.

Here, we present *splitcode* which introduces versatile new features for general preprocessing needs. *splitcode* is a flexible solution with a low memory and computational footprint that can reliably, efficiently, and error-tolerantly preprocess technical sequences based on a user-supplied structure of how those sequences are organized within reads. For example, *splitcode* can simultaneously trim technical sequences, parse combinatorial barcodes that are variable in length and inconsistent in location within a read, and extract UMIs that are defined in location with respect to other technical sequences rather than at a set position within a read. These features make *splitcode* a suitable tool for processing variable length staggers at the start of reads; such staggers are often introduced to enhance nucleotide diversity during the early cycles of sequencing, preventing monotemplate issues that would arise from sequencing identical nucleotides during those cycles. The technical sequences that *splitcode* may be useful for identifying include not only barcodes or UMIs but also ligation linkers, integrase attachment sites, and Tn5 transposase mosaic ends. Moreover, *splitcode* can seamlessly interface with other command-line tools, including other read sequencing read preprocessors as well as read mappers, by streaming the pre-processed reads into those tools. Thus, *splitcode* can eliminate the need to write an entirely new file to disk at every step of preprocessing, a practice that currently results in inefficient use of time and disk space. Furthermore, *splitcode* can stream reads into itself, enabling multiple preprocessing steps to be performed in sequence for more complicated assays.

## 2 Materials and methods

### 2.1 Tag sequence identification

Each sequence in the *splitcode* config file along with all sequences within the sequence’s allowable hamming distance and/or indel error tolerance is indexed in a hash map. Each sequence is associated with the tag(s) from which it originated. Reads in FASTQ files are scanned from start to end to identify tags based on hash map lookups. Additionally, users can specify locations and conditions within which a specific tag may appear and only tags satisfying such conditions are identified. Further, by restricting tag identification to only specific regions of reads, the number of hash map queries is reduced therefore improving runtime.

### 2.2 Final barcode sequences

Each combination of tags is assigned a numerical ID, which begins at 0 and is incremented for every newly encountered combination. Each numerical ID, a 32-bit unsigned integer, can be converted to a unique 16-bp final barcode sequence by mapping each nucleotide to a 2-bit binary representation as follows: A = 00, C = 01, G = 10, T = 11. It follows that the numerical ID can be represented in nucleotide-space based on the integer’s binary representation. For example, the numerical ID 0 is AAAAAAAAAAAAAAAA, the numerical ID 1 is AAAAAAAAAAAAAAAT, and the numerical ID 30 is AAAAAAAAAAAAACTG. This interconversion between numerical IDs and nucleotide sequences facilitates simplifying complex barcodes.

### 2.3 Software

The *splitcode* software is written in C++11 and is freely available and open source under the BSD-2 clause license. The framework for *splitcode* is a C++ header file making the direct incorporation of *splitcode* into a software project that involves processing sequencing reads possible. The GUI for the software is implemented as an HTML webpage and uses Emscripten for compilation of the software to WebAssembly. No new data were generated or analyzed for this article describing the *splitcode* software. Documentation for the software is available at https://splitcode.readthedocs.io/.

## 3 Results

### 3.1 Framework and usage

**Figure 1.**
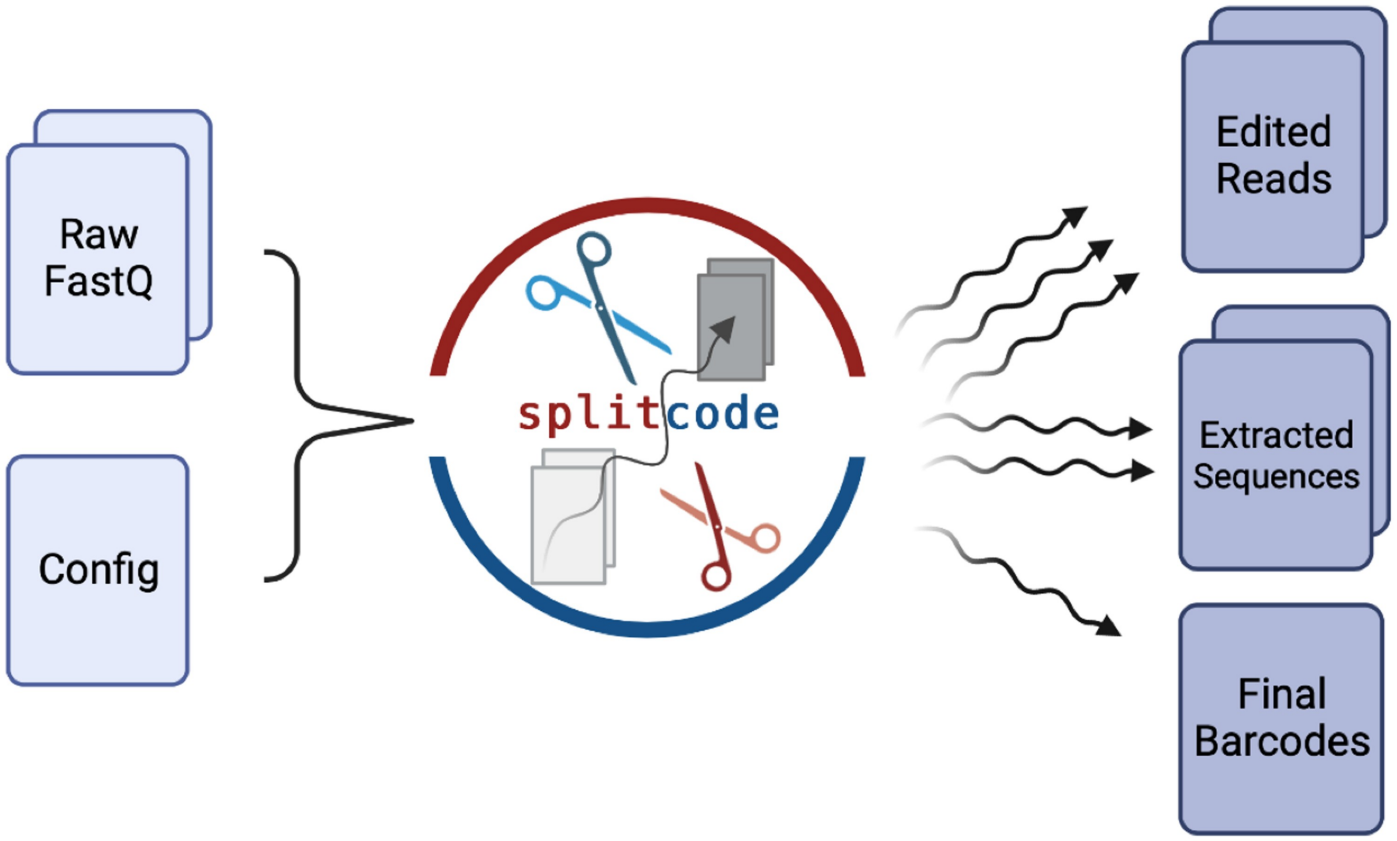
Overview of the *splitcode* workflow. The *splitcode* program takes in a set of FASTQ files and a user-specified config file, which serves as a recipe describing how the reads should be parsed. The user executes *splitcode* on the command-line, specifying command-line options on how the output should be formatted. The output consists of one or more of the following: the original FASTQ files (possibly edited), the extracted sequences (e.g. UMI sequences which are unknown and need to be extracted by using location information or anchor points), and the final barcodes which are unique for each combination of identified tags. The output may take the form of FASTQ files, gzip-compressed FASTQ files, BAM files, or interleaved sequences directed to standard output, depending on what the user specifies.

We refer to the synthetic constructs, or technical sequences that can be identified in reads as tags. Tags are described in the *splitcode* config file with several parameters including a tag ID, the sequence itself, the error-tolerance for identifying that tag, and options such as where the tag might be found within sequencing reads and conditions under which the tag should be searched for. A collection of tags forms a barcode, which can be used to demultiplex reads according to the tags identified within a read. Within the config file, a user can also specify extraction options to delineate how certain subsequences within reads should be extracted. Subsequences can be extracted by using tags as anchor points or can be extracted at user-defined positions within reads. This feature is particularly useful for unique molecular identifier (UMI) sequences which are generally unknown sequences that exist at defined locations within reads. Additionally, in the config file, a user can specify read editing options including trimming and whether identified tags should be replaced with a particular sequence. Thus, identified technical sequences can be modified or trimmed *in situ*. Taken together, this array of options makes it possible for *splitcode* to parse data from a large variety of sequencing assays, including those with many levels of multiplexing (Fig. 1).

**Figure 2.**
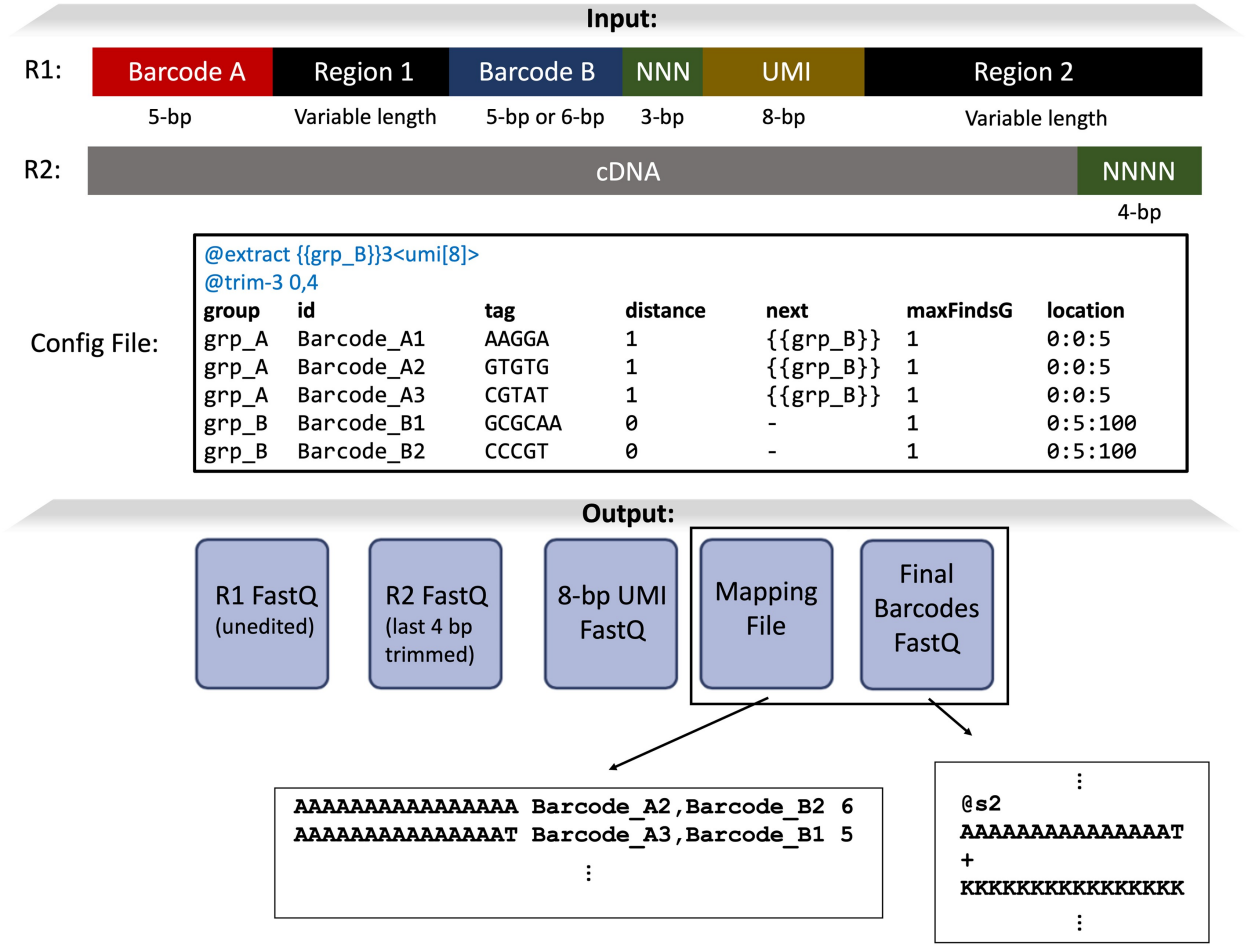
Example of *splitcode* usage. The structure of the reads from this hypothetical sequencing technology contains multiple regions that need to be parsed, including some of variable length. In the config file, each region that needs to be parsed is organized into groups and each “group” contains multiple tags. The tags in the grp_A group have the value 1 in the “distance” column, meaning a hamming distance 1 error tolerance. The values in the “next” column indicate that after a grp_A tag (i.e. Barcode_A1, Barcode_A2, or Barcode_A3) is found, we should next search only for tags in the grp_B group. The “maxFindsG” values of 1 mean that the maximum number of times a specific group can be found is 1 (e.g. after finding a tag in grp_A, stop searching for tags in grp_A). The “location” for grp_A tags have the value 0:0:5, meaning that the tag is found in file #0 (i.e. the R1 file) within positions 0–5 of the read; for grp_B tags, splitcode searches file #0 within positions 5–100. In the header of the config file, the @extract option contains an expression indicating that we should extract an 8-bp sequence, which we name umi, 3 bases following identification of a grp_B tag. The supplied @trim-3 option means that only 3′-end trimming of 0 bases and 4 bases of the R1 file and the R2 file, respectively, should be performed. Thus, here, the output R1 file will contain the original R1 sequences (i.e. the entirety of Barcode A, Region 1, Barcode B, NNN, UMI, and Region 2) while the output R2 file will contain just the cDNA. The output “Final Barcodes” FASTQ file will contain a sequence uniquely identifying a combination of tags and the mapping file allows us to map the final barcode sequence back to the tag combination (the numbers in the right-most column of the mapping file represent how many reads that tag combination was found in). Finally, it is important to note that this is simply one of many ways to parse this read structure with splitcode and users can configure the options how they see fit. Further, users can also customize the output options. For example, users can choose to output reads that contain both grp_A and grp_B tags into one set of files and direct all other reads into a separate set of files, and users can choose whether to output the 8-bp UMI sequence into an independent file or to put it in the FASTQ header of the outputted reads. Users also have the option to output reads as a BAM file with the 8-bp UMI sequence encoded in a SAM tag.

Following construction of the config file (Fig. 2), users can supply the config file to the *splitcode* program on the command-line. Users can further specify the output options for how the final barcode, the (possibly edited) reads, the extracted subsequences should be outputted. The program presents many options for outputting reads, allowing seamless integration with many downstream tools. Importantly, the output can be interleaved and directed to standard output, which can then be directly piped into tools (including *splitcode* itself if another round of read processing is needed) that support such input. This feature makes it possible to send processed reads directly to a read mapper, therefore eschewing the inefficiencies of creating large intermediate files on disk.

### 3.2 Capabilities

The *splitcode* program has many options, some of which can be supplied in the config file and others of which (namely the output options) must be supplied on the command line. In the config file, a user can specify “sequence identification” options for finding tags in reads as well as editing reads *in situ* based on identified tags as well as “read modification and extraction” options for general read trimming and extracting UMI-like sequences. The latter option group is supplied in the header of the config file while the “sequence identification” options are supplied as tab-separated values in a tabular format in the file, an example of which is shown in Fig. 2. A list of some of the *splitcode* config file options is exhibited in Supplementary Table S1.

A graphical user interface (GUI) for *splitcode* facilitates the usage of *splitcode* (Supplementary Fig. S1). This GUI exists as a web page and helps a user create a config file which can then be downloaded. Additionally, this GUI enables live testing of configuration options on user-supplied sample sequences.

Finally, *splitcode* is efficient software: On 150-bp paired-end reads in gzip FASTQ format, *splitcode* can reach throughputs exceeding 10 million reads per minute with memory usage on the order of a few hundred megabytes on a standard laptop, although these performance results vary depending on the task at hand.

## 4 Discussion

The preprocessing of FASTQ files is an important first step in bioinformatics pipelines. This step is frequently inefficient, involving multiple steps with the creation of large intermediate files or writing and running of custom unoptimized scripts which can be challenging with large-scale sequencing data. *splitcode* alleviates some of these inefficiencies via a modular and flexible design to effectively and efficiently handle intricate, hierarchical read structures produced by technologies with many layers of multiplexing. While many of *splitcode’*s features overlap with those of existing bioinformatics software, *splitcode* is not intended to fully recapitulate all the features of existing tools or to replace or outperform any one tool. Rather, *splitcode* is intended to serve as one additional, flexible and versatile tool in a bioinformatics arsenal, and has been designed to be interoperable with other tools. *splitcode* operates not as an alignment algorithm, but on a principle of dictionary lookups. In this approach, technical sequences along with their permissible mismatches are cataloged in a hash table. This makes *splitcode* apt for scenarios requiring identification, interpretation, and modification of short sequences within reads, and it effectively manages extensive lists of lookup sequences. Algorithms like cutadapt which use dynamic programming score matrix to optimize alignment, are more suitable for cases, such as general adapter trimming, that require finding the best possible alignment between two sequences or for finding long technical sequences (in which case, storing the allowable mismatches in a hash table is computationally infeasible). We anticipate that *splitcode* will be used in tandem with other preprocessing tools to provide an effective solution for many bioinformatics needs. Furthermore, we expect that *splitcode* will continue to expand in functionality based on user feedback, user needs, and possibly the introduction of more complicated read structures that may arise from the development of novel sequence census assays.

## Supplementary Material

btae331_Supplementary_Data
