# Supplementary material for: Flexible parsing, interpretation, and editing of technical sequences with splitcode
Source: Bioinformatics. 2024 Jun 14;40(6):btae331. doi: 10.1093/bioinformatics/btae331 (PMC11193061; doi:10.1093/bioinformatics/btae331)
Supplement: btae331_Supplementary_Data [file btae331_supplementary_data.zip › Supplementary_Table_S1.pdf]

Supplementary Table S1:

**Option**	**Description**	**Additional info**
**Sequence Identification Options**		
tag	Tag sequence	String of ATCG bases. Alternately, can supply a file containing multiple tag sequences.
id	Tag name/ID	
distance	Allowable error tolerance	Supports setting hamming distance allowance, indel allowance, and total error (hamming+indel) allowance.
location	Where a tag should be searched for in a read	Can specify file, start position, and end position.
group	Tag group name/ID	Tags can be grouped together under a group name
minFinds	Minimum number of times a tag must be found in a read	If this isn't met, the read is discarded
minFindsG	Minimum number of times a tag group must be found in a read	If this isn't met, the read is discarded
maxFinds	Maximum number of times a tag can be found in a read	Once this is reached, the program simply stops looking for that tag
maxFindsG	Maximum number of times a tag group can be found in a read	Once this is reached, the program simply stops looking for any tag belonging to that group
left	Whether the tag should be a left trimming point	At the location the tag is found, that tag and all bases to the left of the tag in the read are removed
right	Whether the tag should be a right trimming point	At the location the tag is found, that tag and all bases to the right of the tag in the read are removed
next	What tag ID or group ID must come after the tag	When the tag is found, only the tag ID or group ID specified as "next" will be searched for
previous	What tag ID or group ID must come before the tag	The tag will not be searched for unless the tag ID or group ID specified as "previous" was found right before
sub	Sequence to substitute tag with when tag is found in read	Note: This is useful for error correction and one can also

		specify substituting the original tag sequence in if an error-ridden version of the tag was found
partial5	Specifies tag may be truncated at the 5′ end	Can specify the minimum number of bases that must match as well as the mismatch frequency. Useful for adapter trimming.
partial3	Specifies tag may be truncated at the 3′ end	Can specify the minimum number of bases that must match as well as the mismatch frequency. Useful for adapter trimming.
**Read Modification and Extraction Options**		
extract	Pattern(s) describing how to extract UMI and UMI-like sequences from reads	Multiple extractions can be specified (e.g. if there are two UMI sequences in the read structure).
trim-5	Number of base pairs to trim from the 5′-end of reads	
trim-3	Number of base pairs to trim from the 3′-end of reads	
filter-len	Filter reads based on length	
qtrim	Threshold for quality trimming	

The features specified in **Supplementary Table S1** relate to read editing and tag identification. Downstream of the tag identification process, there are more options to further process identified tags. For example, using the --keep and --keep-grp command-line options, a user can specify combination(s) of tags or tag groups that should be retained and only those reads will be kept. Likewise, using the --discard and --discard-grp command-line options, a user can specify a combination(s) that should be discarded and those reads will be discarded. Furthermore, using the --keep and --keep-grp options, a user can specify specific combinations to be outputted into specific files, enabling demultiplexing based on barcodes or barcode combinations.
