# Supplementary figures and images for: Flexible parsing, interpretation, and editing of technical sequences with splitcode
Source: Bioinformatics. 2024 Jun 14;40(6):btae331. doi: 10.1093/bioinformatics/btae331 (PMC11193061; doi:10.1093/bioinformatics/btae331)

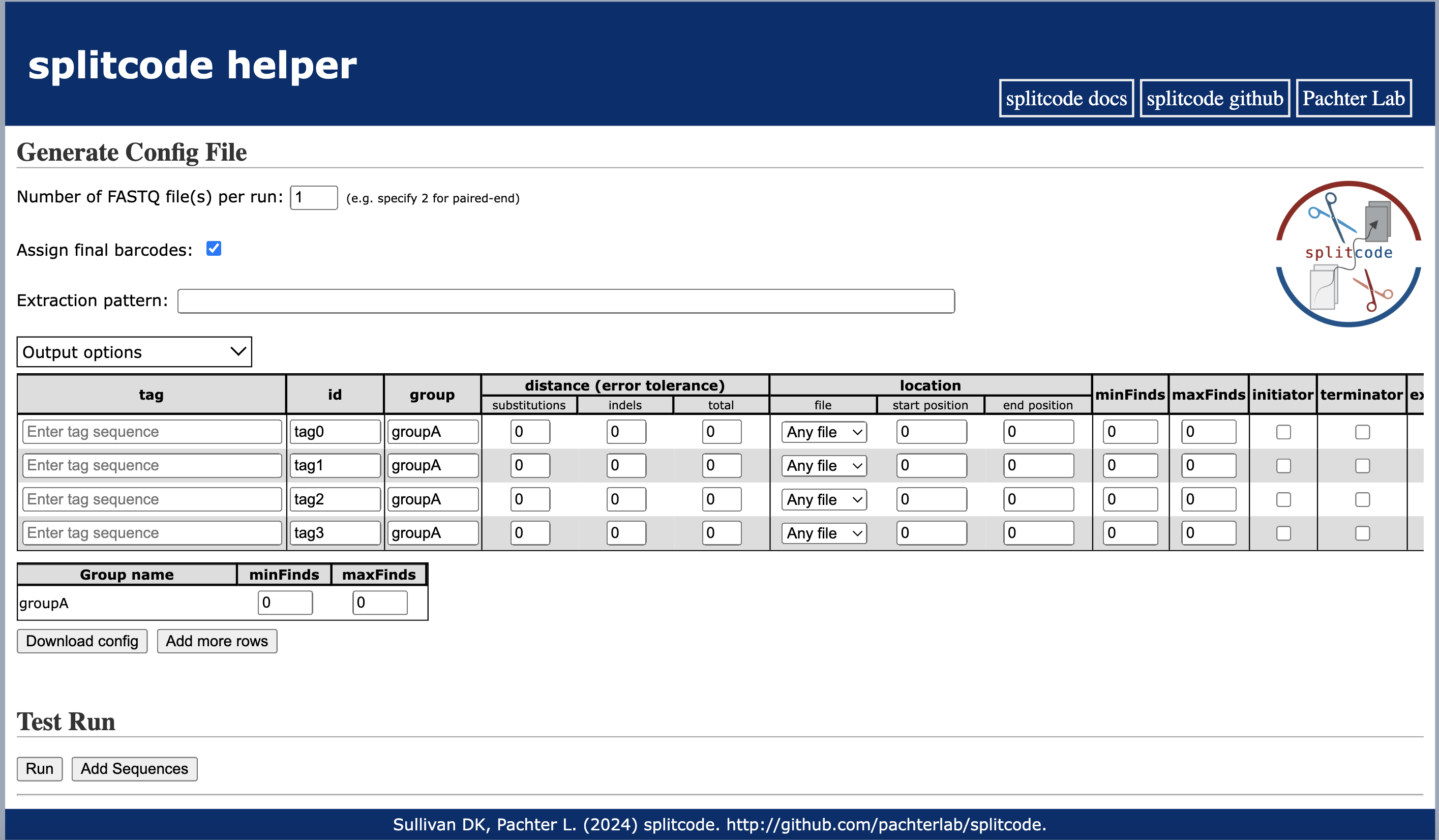

Supplement: btae331_Supplementary_Data [file btae331_supplementary_data.zip › Supplementary_Fig_S1.png]
